# The Empiric

**Published:** 1869-02

**Authors:** 


### The Empiric.

We have all of us, doubtless, enjoyed the reading of *Hudibras*, and perchance have floating in our minds many a line or couplet thence derived, to point a moral if not to adorn a tale. But probably even all physicians have not read the admirable sketch of the *Empiric*, by the same author. Those who now read it will see that the character is drawn to the life, for the quack is essentially the same now as in Butler's day.

The *Empiric*, says this author, is a medicine-monger, probationer of receipts, and doctor of medicine; he is perpetually putting his medicines upon their trial, and very often finds them guilty of manslaughter, but still they have some trick or other to come off, and avoid burning by the hand of the hangman. He points to his trials of skill, and challenges death at so many weapons, that though he is sure to fail at every one, he cares not; for if he gets money, he is sure to get off. For it is but posting up diseases for poltroons in all the public places of the town, and daring them to meet him again, and his credit stands as fair with the rabble as ever it did. He makes nothing of the pox, and the running of the reins, but will undertake to cure them, and tie one hand behind him, with so much ease and safety that his patients may surfeit and be drunk as often as they please, and follow their business, that is, whores and him, without any inconvenience to their health and occasions; and he cures with so much secrecy that they shall never know how it came about. He professes no cure no pay, as well he may, for if nature does the work he is paid for it; if not, he neither wins nor loses, and, like a cunning rook, lays his bet so artfully that, let the chance be what it will, he either wins or saves. He cheats the rich for their money and the poor for charity, and if either succeeds both are pleased, and he passes for a very just and conscientious man, for as those that pay nothing ought at least speak well of their entertainment, their testimony makes way for those that are able to pay for both. He finds he has no reputation among those who know him, and fears he is never likely to have, and therefore posts up his bills to see if he can thrive better among those that know nothing of him.

He keeps his post continually, and will undertake to maintain it against all the plagues of Egypt. He sets up his trade on a pillar or the corner of a street; these are his ware-houses, where all he has is to be seen, and a great deal more, for he that looks further finds nothing at all.—*Druggists' Circular.*

